# Systemic–retinal inflammatory crosstalk in diabetic macular edema: correlation between hematologic indices and macular OCT-features

**DOI:** 10.1186/s40942-025-00794-y

**Published:** 2026-01-13

**Authors:** Ohisa Harley, Yufilia Suci Amelia, Elsa Gustianty, Nanny N. M. Soetedjo, Arief S. Kartasasmita

**Affiliations:** 1 Doctoral Program in Medical Sciences, Faculty of Medicine, Padjadjaran University, Bandung, West Java Indonesia; 2 Netra Eye Clinic Centre, Bandung, West Java Indonesia; 3 Unpad Hospital, Jatinangor, West Java Indonesia; 4 Departement of Ophthalmology, Faculty of Medicine, Padjadjaran University, Bandung, West Java Indonesia; 5 Cicendo National Eye Hospital Center, Bandung, West Java Indonesia; 6 Departement of Endocrinology and Internal Medicine, Faculty of Medicine, Padjadjaran University, Bandung, West Java Indonesia

**Keywords:** Diabetic macular edema, Diabetic retinopathy, Inflammation, Markers, Optical coherence tomography

## Abstract

**Purpose:**

This study investigated the relationship between systemic inflammatory indices and optical coherence tomography (OCT)–derived retinal inflammatory biomarkers in patients with diabetic macular edema (DME) associated with non-proliferative diabetic retinopathy (NPDR).

**Methods:**

A cross-sectional study was conducted on 40 eyes from patients with clinically significant DME. OCT biomarkers, including hyperreflective foci (HRF), subretinal fluid (SRF), and hard exudates, were evaluated. Systemic inflammatory markers—neutrophil-to-lymphocyte ratio (NLR), monocyte-to-lymphocyte ratio (MLR), platelet-to-lymphocyte ratio (PLR), and systemic immune-inflammation index (SII)—were derived from complete blood counts. Correlations between these parameters were analyzed using non-parametric tests.

**Results:**

A total of 40 eyes from 40 patients with NPDR with DME were analyzed. The mean central macular thickness was 396.28 ± 124.75 μm. Systemic inflammatory markers (NLR, MLR, PLR, SII) showed no significant differences between mild and severe DME groups. Among OCT biomarkers, eyes with hard exudates demonstrated significantly higher PLR values (*p* = 0.018), while differences in NLR, MLR, and SII were insignificantly higher in HRF > 30 and positive hard exudates.

**Conclusions:**

DME pathogenesis appears to involve two interacting inflammatory pathways: systemic platelet-driven vascular inflammation and localized microglial-mediated neuroinflammation. Elevated PLR reflects systemic endothelial injury and correlates with vascular leakage, whereas OCT features, such as HRF and SRF, represent compartmentalized retinal inflammation. Integrating systemic hematologic indices with OCT inflammatory biomarkers offers a practical and translational framework for assessing inflammatory activity in DME and may guide personalized monitoring and therapeutic strategies.

## Introduction

Diabetic macular edema (DME) is a leading cause of vision loss in diabetic retinopathy (DR), resulting from chronic hyperglycemia, oxidative stress, and inflammation that disrupts the blood–retina barrier (BRB) [1–3]. Although vascular endothelial growth factor (VEGF) plays a central role in its development, increasing evidence suggests that DME represents a chronic inflammatory disorder characterised by endothelial dysfunction, microglial activation, and immune dysregulation within the retinal neurovascular unit [4–8]. 

Optical coherence tomography (OCT) allows non-invasive visualization of retinal structural changes associated with inflammation, including hyperreflective foci (HRF), subretinal fluid (SRF), and hard exudates. HRF reflect clusters of activated microglia and macrophages, SRF indicates retinal pigment epithelium (RPE) dysfunction and outer BRB breakdown, while hard exudates signify chronic vascular leakage and lipid accumulation [9–13]. These OCT-derived features are increasingly recognized as structural biomarkers of retinal inflammation and vascular permeability in DME.

Systemic inflammation has also been implicated in the pathogenesis of DR and DME. Hematologic indices such as the neutrophil-to-lymphocyte ratio (NLR), monocyte-to-lymphocyte ratio (MLR), platelet-to-lymphocyte ratio (PLR), and systemic immune-inflammation index (SII) have emerged as accessible and cost-effective indicators of systemic immune activation [14–21]. While these markers have been associated with DR severity, their relationship with localized inflammatory manifestations visualized on OCT in DME remains poorly characterized. Understanding this interaction is clinically relevant because systemic inflammatory activity may influence disease chronicity, predict response to anti-VEGF therapy, and help stratify patients at higher risk of persistent or refractory macular edema. Clarifying the interplay between systemic and retinal inflammation could therefore enhance individualized management strategies and support early intervention to prevent irreversible visual loss.

To our knowledge, this is the first study to correlate peripheral inflammatory indices with OCT-derived inflammatory biomarkers specifically in non-proliferative diabetic retinopathy (NPDR)–associated DME. This study aimed to investigate the correlation between systemic inflammatory blood markers (NLR, MLR, PLR, and SII) and OCT-based retinal biomarkers (HRF, SRF, and hard exudates) in patients with DME within the NPDR spectrum. By integrating systemic hematologic and imaging parameters in a homogeneous cohort, this study seeks to provide new insights into the inflammatory continuum linking systemic immune imbalance and localized retinal pathology, with potential implications for both prognosis and therapeutic decision-making in DME.

## Methods

### Study design

This was a single-center, cross-sectional, comparative study between March and August 2025 at Netra Eye Clinic. The study adhered to the tenets of the Declaration of Helsinki and received approval from the Ethics Committee of Universitas Padjadjaran (No. 85/UN6.KEP/EC/2025). Written informed consent was obtained from all participants prior to enrollment.

### Study population

A total of 40 eyes from 40 patients with type 2 diabetes mellitus (T2DM) and clinically significant diabetic macular edema (DME) were consecutively recruited. The inclusion criteria were: (1) age > 18 years, (2) confirmed diagnosis of T2DM by an internist, and (3) non-proliferative diabetic retinopathy (NPDR) with DME confirmed by OCT. Exclusion criteria included: (1) any acute systemic or ocular inflammatory disease, (2) autoimmune or immune-mediated disorders, (3) renal failure requiring dialysis or transplant, (4) recent major surgery within one month, and (5) prior ocular intervention such as panretinal photocoagulation, vitrectomy, or intravitreal anti-VEGF therapy within the past month.

### Ophthalmic examination

All participants underwent comprehensive ophthalmic evaluation by a vitreoretinal specialist, including best-corrected visual acuity (BCVA), slit-lamp biomicroscopy, intraocular pressure measurement, fundus photography (CLARUS 500, Zeiss), and spectral-domain optical coherence tomography (SD-OCT; CIRRUS 5000, Zeiss). OCT imaging was performed after pharmacologic pupil dilation using standardized horizontal and vertical raster scans centered on the fovea. Central macular thickness (CMT) was automatically calculated as the distance between the inner limiting membrane and the retinal pigment epithelium. DME was defined as CMT ≥ 300 μm. Two masked graders independently reviewed all scans; discrepancies were resolved by consensus. The diagnosis of NPDR were based on Early Treatment Diabetic Retinopathy Study (ETDRS).

OCT inflammatory biomarkers recorded included:

- Hyperreflective foci (HRF): HRF were distinct punctate foci with reflection intensity equal to or greater than the nerve fiber layer, distributed across retinal layers. HRF count was manual from inner limiting membrane to RPE; and categorized as < 30 or ≥ 30 foci [13, 22].

- Subretinal fluid (SRF): hypo-reflective area under the photoreceptor layer, with or without serous detachment [23].

- Hard exudates: hard exudates have larger diameter compared to HRF (greater than 4 mm), higher reflectivity (reflectivity similar to RPE layer), and the presence of back-shadowing [23].

Each OCT feature was classified semi-quantitatively and verified by a vitreoretinal consultant blinded to clinical and laboratory data.

### Laboratory evaluation

Venous blood samples (5 mL) were drawn from all participants on the same day as ocular examination. Routine laboratory tests included complete blood count, fasting blood glucose, and HbA1c. All analyses were performed at an accredited central laboratory using an automated hematology analyser. Hematologic inflammatory markers were calculated as follows:

- NLR: neutrophil/lymphocyte ratio
- MLR: monocyte/lymphocyte ratio
- PLR: platelet/lymphocyte ratio
- SII: (neutrophil × platelet) / lymphocyte count

### Data collection and outcome measures

Baseline demographic and clinical variables—age, sex, diabetes duration, comorbidities, and systemic treatment history—were obtained via medical records and interviews. The primary outcome was the correlation between systemic inflammatory indices (NLR, MLR, PLR, SII) and OCT-based inflammatory markers (HRF, SRF, and hard exudates). Secondary outcomes included associations between these markers and the severity of macular thickening (mild: CMT < 350 μm; severe: CMT ≥ 350 μm) [24]. The OCT parameter and DME classification were assessed blindly and randomly by two vitreo-retina specialists.

### Statistical analysis

All data were analyzed using SPSS version 26.0 (IBM Corp., USA). Continuous variables were expressed as mean ± standard deviation or median (interquartile range) as appropriate, while categorical variables were summarized as frequencies and percentages. Normality was tested with the Shapiro–Wilk test. Differences between groups were analyzed using an independent t-test or Mann–Whitney U-test for continuous variables and a Chi-square or Fisher’s exact test for categorical variables. A p-value < 0.05 was considered statistically significant.

## Results

### Baseline characteristics

**Fig. 1:**
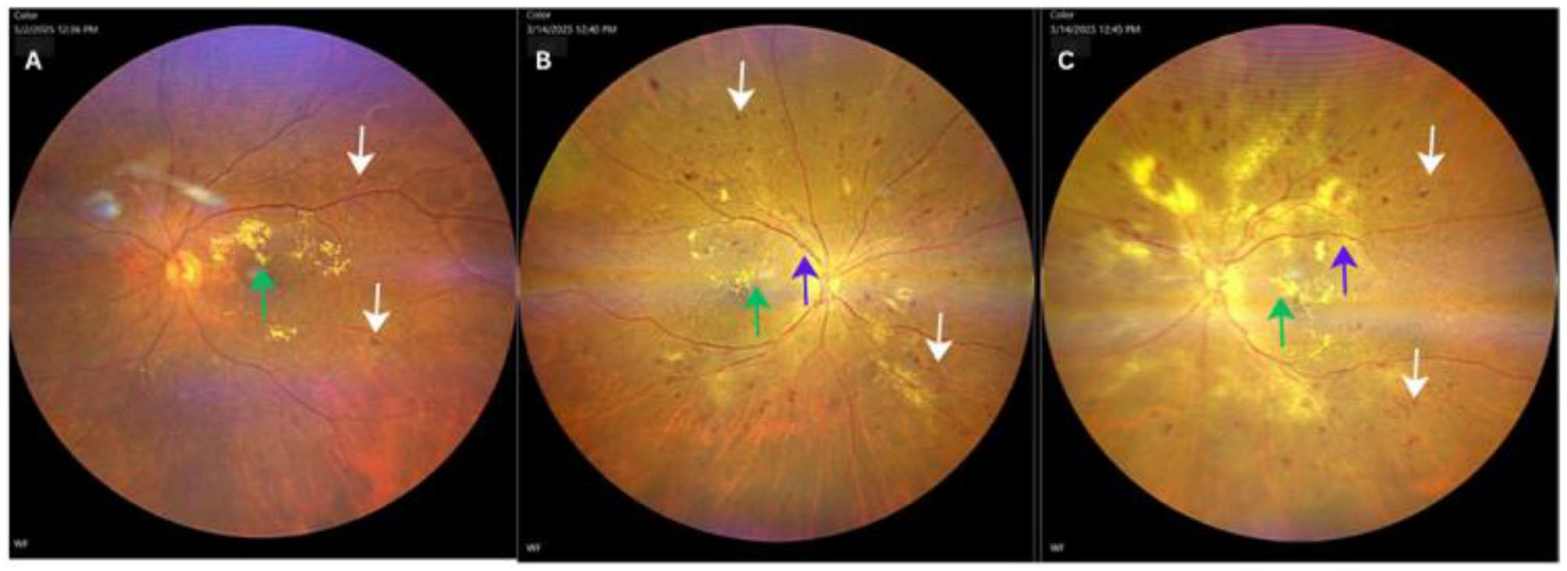
Fundus photographs of NPDR. (**A**) Mild-moderate NPDR showing microaneurysms (white arrow) and hard exudates (green arrow). (**B**–**C**) Severe NPDR demonstrating multiple microaneurysms across all quadrants (white arrow), hard exudates (green arrow), venous beading (blue arrow). *NPDR= Non-proliferative diabetic retinopathy*

**Fig. 2:**
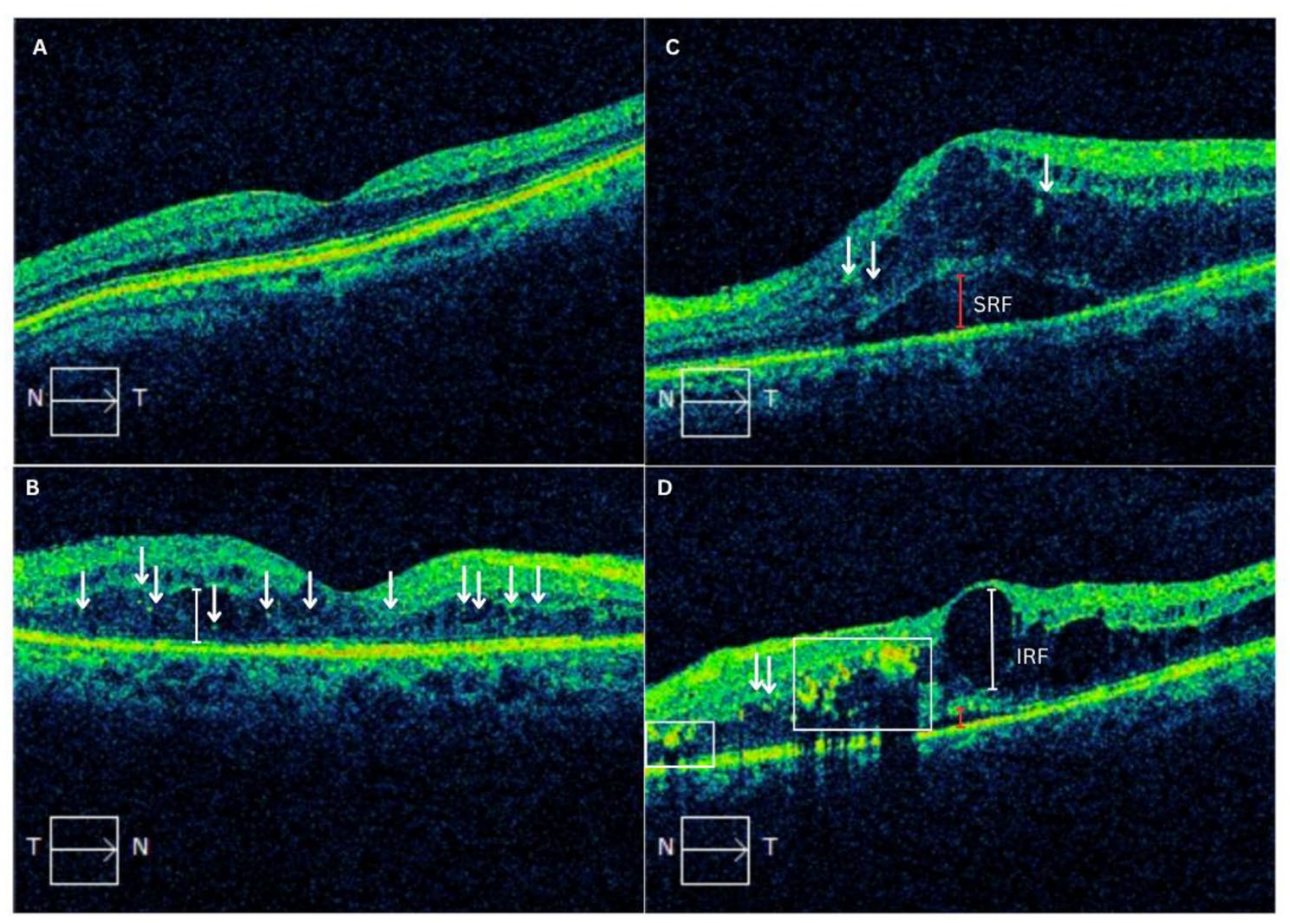
Representative macular OCT scans demonstrating the spectrum of DME. (**A**) Normal foveal contour with intact retinal architecture and no fluid accumulation. (**B**) Mild DME showing subretinal fluid (IRF; white vertical line) and scattered hyperreflective foci (white arrows) within the inner retina. (**C**–**D**) Severe DME exhibiting intraretinal cystoid spaces (IRF; white vertical line) and clustered hyperreflective foci (white arrows), representing localized inflammatory or lipid aggregates. *OCT = optical coherence tomography; DME = diabetic macular edema; SRF = subretinal fluid; IRF = intraretinal fluid*

A total of 40 eyes from 40 participants with NPDR were enrolled in the final analysis, with a mean age of 59.5 ± 8.20 years, consisting of 17 male and 23 female participants. The severity of NPDR included 34 patients (85%) with severe NPDR and 6 patients (15%) with mild-to-moderate NPDR. Our participants have undergone several treatments, including two patients who received anti-VEGF therapy and eight patients who received PRP laser. The baseline characteristics data of participants are presented in Table 1. The CMT baseline demonstrated a statistically significant difference in the severe DME compared to mild-moderate DME (476.86 ± 119.54 μm vs. 307.21 ± 43.51 μm, *p* = 0.00). The characteristics of DME in OCT parameter were described in Table 2. The fundus photograph and OCT macula characteristics were described in Figs. 1 and 2. Furthermore, patients were divided into two groups: Mild DME (*n* = 19) and Severe DME (*n* = 21). The NLR, PLR, MLR, and SII were not statistically different among groups. (Table 3.)

**Table 1:** Baseline characteristics of studies

Variable	Total of DME *N* = 40	Mild DME *N* = 19	Severe DME *N* = 21	*P* value
Age (year)	59.5 ± 8.20	60.79 ± 6.51	57.92 ± 10.16	0.25
Sex				
Male	17			
Female	23			
Visual acuity (logMar)	0.53 ± 0.40	0.44 ± 0.26	0.62 ± 0.49	0.10
Intraocular pressure (mmHg)	15.25 ± 3.01	15.57 ± 2.70	17.62 ± 3.15	0.18
Duration of diabetes (year)	9.35 ± 5.63	8.64 ± 5.12	8.69 ± 4.62	0.16
BMI (kg/m^2^)	24.64 ± 3.00	23.70 ± 2.94	25.66 ± 2.82	0.09
CMT (µm)	396.28 ± 124.75	307.21 ± 43.51	476.86 ± 119.54	0.00
Severity of NPDR				
Mild-Moderate	6			
Severe	34			
History of treatment				
anti VEGF intravitreal	2			
PRP Laser	8			
History of systemic disease				
Hypertension	21			
Nephropathy	2			
Neuropathy	0			
Cardiovascular disease	3			
Free blood glucose (mg/dl)	184.53 ± 63.02	188.58 ± 66.52	180.86 ± 61.09	0.70
HbA1C (%)	8.05 ± 2.19	8.19 ± 2.31	7.92 ± 2.13	0.70
Blood count cells				
Platelet	309,692.96 ± 84,421.32	314,789.47 ± 149,402.72	331,904.76 ± 92,971.45	0.215
Neutrophil absolute count	5,741.15 ± 1,721.40	5,015.21 ± 1,535.34	6,623.31 ± 1,820.36	0.26
Leukocyte absolute count	8,935 ± 2,269.48	7,957.14 ± 1,985.60	10,238.46 ± 2,444.22	0.11
Lymphocytes absolute count	2,841.33 ± 841.62	2,659.79 ± 826.59	3,281.15 ± 871,39	0.103
Monocyte absolute count	362.65 ± 218.61	282,14 ± 150.70	365.15 ± 218.77	0.10

BMI = Body Mass Index; NPDR = Non-proliferative diabetic retinopathy; PRP = Panretinal photocoagulation; VEGF = Vascular Endothelial Growth Factor; HbA1c = Glycated hemoglobin

**Table 2:** Characteristics of OCT biomarkers in DME

OCT Biomarker	Mild DME *N* = 19	Severe DME *N* = 21	*P* value
HRF			
< 30	4	4	0.874
> 30	15	17	
IRF			
Negative	0	0	N/A
Positive	19	21	
SRF			
Negative	13	14	0.906
Positive	6	7	
Hard exudates			0.270
Negative	2	5	
Positive	17	16	

HRF = Hyperreflective focci; IRF = intraretinal fluid; SRF = sub-retinal fluid

N/A due to the absence of comparison groups (IRF present in all cases)

**Table 3:** Systemic inflammatory markers in DME

Variable	Total *N* = 40	Mild DME *N* = 19	Severe DME *N* = 21	*p* value ^+++^
NLR	2.16 ± 0.86	2.28 ± 1.00	2.07 ± 0.73	0.424
MLR	0.13 ± 0.08	0.13 ± 0.07	0.14 ± 0.09	0.892
PLR	124 ± 58.97	133.73 ± 71.56	115 ± 44.72	0.357
SII	712,926.27 ± 451,533.51	757,342.15 ± 567,871.44	681,788.09 ± 324,341.53	0.968

DME = diabetic macular edema; NLR = neutrophil-to-lymphocyte ratio; MLR = monocyte-to-lymphotcyte ratio; PLR = platelet-to-lymphocyte-ratio; SII = systemic immune inflammation index

### The relationship between systemic inflammatory markers and OCT biomarkers

For the group with HRF ≥ 30, the values of PLR, MLR, and SII were higher compared to the HRF > 30 group, although the differences were not statistically significant (*p* = 0.073, *p* = 0.171, and *p* = 0.063, respectively). In the SRF subgroup, there were no significant differences in NLR, PLR, MLR, or SII values between eyes with and without SRF (all *p* > 0.05).

Conversely, in the hard exudates subgroup, the PLR value was significantly higher in eyes with hard exudates compared to those without (*p* = 0.021), while NLR and SII values tended to be higher in the hard exudate group but did not reach statistical significance. (Table 4)

**Table 4:** The correlation between blood inflammatory markers and OCT marker

OCT Parameter	*n*	NLR	PLR	MLR	SII
HRF > 30	8	2.2 ± 0.90	131.78 ± 62.73	0.14 ± 0.08	766,323.12 ± 480,304.10
HRF < 30	32	1.98 ± 0.70	92.87 ± 23.75	0.10 ± 0.05	499,338.87 ± 221,607.58
*P value*		0.539	0.076	0.174	0.065
SRF +	13	2.06 ± 0.86	119.46 ± 55.87	0.12 ± 0.06	663,515.38 ± 402,750.15
SRF -	27	2.22 ± 0.87	126.18 ± 61.32	0.14 ± 0.09	736,716.70 ± 478,696.37
*P value*		0.458	0.798	0.798	0.549
Hard exudates +	33	2.21 ± 0.79	128.69 ± 56.20	0.14 ± 0.08	732,680.03 ± 432,559.65
Hard exudates -	7	1.93 ± 1.18	101.85 ± 71.16	0.10 ± 0.06	619,801.42 ± 561,305.65
*P value*		*0.110*	0.018*	*0.293*	*0.075*

HRF = Hyperreflective focci; IRF = intraretinal fluid; SRF = sub-retinal fluid;

*p value < 0.05, non-parametric data presented in mean ± standard deviation

## Discussion

The present study investigated the relationship between systemic inflammatory indices and OCT-derived retinal inflammatory biomarkers in patients with DME. Among the hematologic markers examined, only the PLR demonstrated a statistically significant association with the presence of hard exudates. In contrast, other systemic markers—including the NLR, MLR, and SII—showed no meaningful correlations with other OCT features such as HRF and SRF. These results suggest that platelet-driven vascular inflammation may play a more direct role in macular vascular leakage. This plausible mechanism is also supported by the fact that platelet activation is known to occur in diabetes and may contribute to microvascular damage [25]. In contrast, other systemic inflammatory processes may not accurately reflect localized retinal inflammation within the macular compartment.

Previous meta-analyses have demonstrated that NLR, PLR, and SII are correlated with the severity of DR, particularly in proliferative disease, where systemic inflammation and ischemia are widespread [21]. However, DME differs from proliferative DR in that it represents a localized manifestation of vascular leakage within the macula rather than a panretinal ischemic process [5, 26]. This distinction may explain the weaker correlations observed between systemic inflammatory indices and OCT biomarkers in our study. The findings highlight that systemic inflammation exerts a limited influence on the localized microenvironment of the macula, where neuroglial activation predominates.

The pathogenesis of DME, however, cannot be fully explained by a single mechanism. It involves two interrelated yet distinct inflammatory pathways—systemic vascular inflammation and local neuroinflammation—that act synergistically to promote macular edema and retinal dysfunction [8, 27]. 

**Fig. 3:**
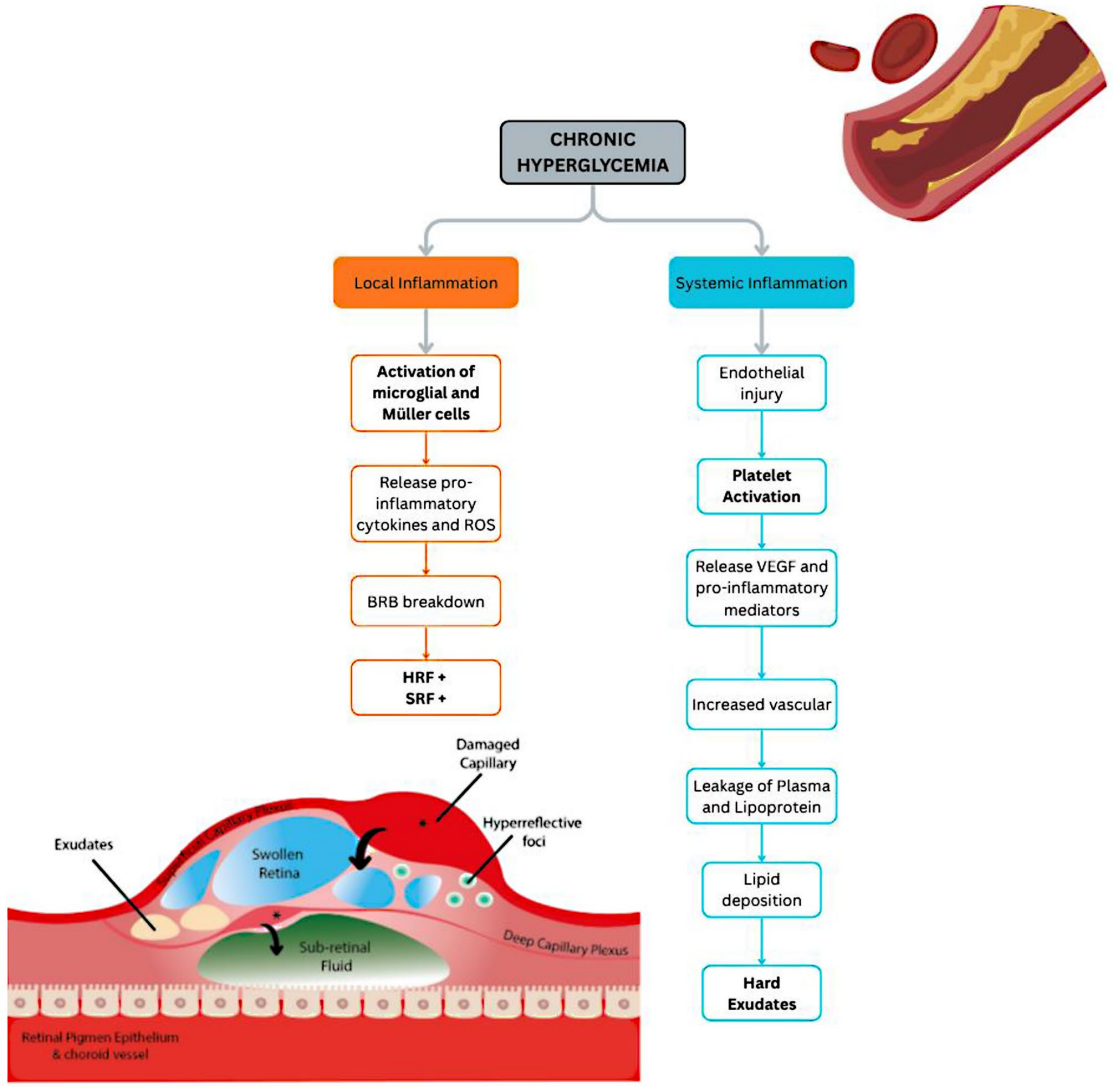
Exploratory Conceptual Framework in our study. Chronic hyperglycemia triggers both local retinal and systemic inflammatory responses that disrupt the blood–retina barrier, promote vascular leakage, and lead to fluid accumulation (HRF and SRF) and hard exudate formation within the macula. HRF = hyperreflective foci; SRF = subretinal fluid; BRB = blood–retina barrier; DME = diabetic macular edema

DME has been widely attributed to endothelial dysfunction, which facilitates vascular leakage and lipid extravasation, with hard exudates representing lipid deposits originating from the vascular lumen. Within this established pathophysiological framework, our findings suggest that the presence of hard exudates may reflect a systemic inflammatory milieu, as indicated by the significant association with PLR. Chronic hyperglycemia and oxidative stress are known to induce endothelial injury and platelet activation in the systemic circulation. Activated platelets release vasoactive and pro-inflammatory mediators—including vascular endothelial growth factor (VEGF), platelet-derived growth factor (PDGF), and thromboxane A₂—that disrupt endothelial junctions and increase vascular permeability. ^27–30^ This cascade promotes plasma and lipoprotein leakage into retinal tissue, leading to lipid deposition and the formation of hard exudates. Hematologically, this state may be reflected by an elevated PLR, which integrates enhanced platelet activity and reduced lymphocyte-mediated immune regulation. In this context, PLR may serve as a surrogate marker of platelet-driven systemic vascular inflammation, consistent with the association observed in this study (Fig. 3).

In contrast, HRF and SRF likely represent predominantly localized retinal inflammatory processes. The local neuroinflammatory pathway arises within retinal tissue itself, where chronic hyperglycemia activates resident microglia and Müller cells, leading to the release of pro-inflammatory cytokines such as IL-1β and TNF-α, as well as reactive oxygen species [31–34]. These mediators contribute to blood–retina barrier disruption, neuronal injury, and glial dysfunction. On OCT imaging, such changes manifest as HRF—thought to represent clusters of activated microglia or macrophages—and SRF, which reflects retinal pigment epithelium dysfunction and outer blood–retina barrier breakdown [13, 35]. Because the retina constitutes an immune-privileged compartment, these localized inflammatory processes may occur relatively independently of systemic immune activity, providing a plausible explanation for the lack of significant correlations between HRF or SRF and systemic inflammatory indices such as NLR, MLR, and SII in the present study.

Importantly, the conceptual framework illustrated in Fig. 3 is not intended to imply causality or provide mechanistic proof [28–30, 36]. Rather, it represents a hypothesis-generating interpretative model derived from existing pathophysiological theories and the observed associations in this cross-sectional analysis. The present findings are intended to provide a conceptual basis for future longitudinal and mechanistic investigations exploring the temporal interplay between systemic inflammation, retinal neuroinflammation, and treatment-resistant or persistent DME.

Furthermore, systemic comorbidities such as hypertension, dyslipidemia, or diabetic nephropathy may modulate NLR and SII values without necessarily reflecting retinal inflammation, introducing biological variability that could mask associations. In contrast, PLR appears more stable, as platelet counts are less affected by transient stress or minor infections compared to neutrophil-based indices. This stability may partly explain why PLR emerged as the most reliable systemic correlate of vascular leakage in this study.

The strength of this study lies in its homogeneous sample, consisting solely of eyes with NPDR and DME. This design minimizes confounding from advanced proliferative disease, allowing for a focused exploration of early inflammatory–vascular interactions within the macula. Moreover, although most associations did not reach statistical significance, consistent trends were observed in which eyes with positive OCT inflammatory markers tended to show higher NLR, PLR, and SII values. The significant association of PLR with hard exudates further supports the potential contribution of platelet-driven inflammatory activity in DME pathophysiology. In addition, the persistence of a significant association between PLR and hard exudates is noteworthy, as hard exudates reflect chronic lipid deposition from prolonged vascular leakage and tend to resolve more slowly than retinal fluid. To the best of our knowledge, this is the first study demonstrating the association between PLR and OCT markers; therefore, this could serve as a foundational step in the development of DME markers.

Despite these strengths, several limitations should be acknowledged. First, our findings should be interpreted carefully due to the single-center nature of the study, which inevitably limits the generalizability of the findings. The small sample size and unequal subgroup distribution, particularly in the hard exudates and HRF categories, may have limited statistical power and rendered some findings, including the PLR association, susceptible to instability. When interpreting these findings, it is important to consider the differential temporal effects of local ocular treatments and systemic inflammatory processes. OCT-derived retinal morphology can be substantially modified by local ocular treatments, whereas systemic inflammatory markers such as PLR are generally considered indicators of chronic, low-grade inflammation that evolve over longer timeframes. Moreover, ocular treatments may lead to structural improvement on OCT without substantially altering systemic inflammatory markers, which could further reduce the ability to detect meaningful associations between systemic inflammation and retinal morphology. Although approximately 25% of eyes had received prior ocular treatment, inclusion of these cases may have attenuated detectable associations between systemic inflammatory markers and OCT features. Therefore, these results should be interpreted with caution. This finding may therefore indicate a systemic inflammatory contributing to chronic or treatment-resistant DME in real-world clinical settings.

The cross-sectional nature of the study also limits causal inference, and the modest sample size may have restricted the detection of subtler associations. Moreover, the consistent directionality of findings supports the biological plausibility of the observed relationships. Future longitudinal studies integrating quantitative OCT metrics, cytokine profiling, and OCT angiography are warranted to validate these findings and further elucidate the temporal dynamics between systemic and local inflammation.

## Conclusion

This study demonstrates that platelet-driven vascular inflammation, reflected by an elevated PLR, is closely linked to vascular leakage and hard exudate formation in DME. In contrast, other systemic markers such as NLR, MLR, and SII showed no significant correlation with OCT inflammatory features, suggesting that localized retinal inflammation is predominantly governed by microglial activation within the immune-privileged retina. Integrating systemic hematologic indices with OCT-based biomarkers provides a simple and practical framework for assessing inflammatory activity in DME. Further prospective studies are warranted to validate this dual-pathway model and its potential clinical applications.

## Data Availability

The datasets used and/or analyzed during the current study are available from the corresponding author upon reasonable request.

## Abbreviations

BCVA: Best Corrected Visual Acuity
BMI: Body Mass Index
BRB: Blood–Retina Barrier
CMT: Central Macular Thickness
DME: Diabetic Macular Edema
DR: Diabetic Retinopathy
HbA1c: Glycated Hemoglobin
HRF: Hyperreflective Foci
IL-1β: Interleukin-1 Beta
IRF: Intraretinal Fluid
MLR: Monocyte-to-Lymphocyte Ratio
NLR: Neutrophil-to-Lymphocyte Ratio
NPDR: Non-Proliferative Diabetic Retinopathy
OCT: Optical Coherence Tomography
PDGF: Platelet-Derived Growth Factor
PLR: Platelet-to-Lymphocyte Ratio
PRP: Panretinal Photocoagulation
RPE: Retinal Pigment Epithelium
ROS: Reactive Oxygen Species
SII: Systemic Immune–Inflammation Index
SRF: Subretinal Fluid
T2DM: Type 2 Diabetes Mellitus
TNF-α: Tumor Necrosis Factor-Alpha
VEGF: Vascular Endothelial Growth Factor
